# Effects of Maternal Peanut Intake and Breastfeeding Duration on Offspring DNA Methylation

**DOI:** 10.1002/fsn3.71129

**Published:** 2025-10-21

**Authors:** Jessica L. Garay, Margaret A. Voss, Stefanie R. Pilkay

**Affiliations:** ^1^ Department of Nutrition and Food Studies Syracuse University Syracuse New York USA; ^2^ School of Social Work Syracuse University Syracuse New York USA

**Keywords:** brain‐derived neurotrophic factor, diet, epigenetics, gene expression, inflammation, interleukin‐6, pregnancy

## Abstract

Emerging evidence suggests that maternal diet during pregnancy and breastfeeding may influence epigenetic modifications in offspring genes related to neurodevelopment and inflammation, although the specific mechanisms and long‐term implications of these effects are not well understood. This study evaluated the influence of maternal peanut intake during pregnancy and/or breastfeeding on the relationships between breastfeeding duration, household income, and child race with DNA methylation of genes associated with inflammation and neurodevelopment. Children aged 2–7 years (*N* = 35) provided saliva samples and their parent/guardian completed questionnaires related to maternal diet during pregnancy and breastfeeding as well as family demographics. We found that maternal consumption of peanuts and peanut butter during pregnancy may enhance the epigenetic sensitivity of the BDNF gene region to breastfeeding duration. Among participants with maternal consumption of both peanuts and peanut butter during pregnancy, longer breastfeeding duration was associated with increased DNAm of BDNF at 12 CpG sites and decreased DNAm at 3 CpG sites. Interestingly, for participants with maternal consumption of peanut butter only, longer breastfeeding duration was associated with decreased BDNF DNAm, suggesting a potential for increased gene expression. A similar pattern emerged when household income was considered. Among mothers who consumed both peanuts and peanut butter during breastfeeding, BDNF DNAm increased with increasing household income. In contrast, no interaction emerged between income and BDNF DNAm when mothers consumed peanut butter only during breastfeeding. Additionally, patterns by race/ethnicity differed depending on maternal diet. Among BIPOC participants, IL6‐AS1 DNAm was lower compared to white participants when mothers consumed both peanuts and peanut butter during pregnancy. However, when mothers consumed only peanut butter, BIPOC participants showed higher IL6‐AS1 DNAm levels compared to their white peers.

## Graphical abstract

Maternal peanut intake during pregnancy moderated the relationship between breastfeeding duration and child DNAm; effects differed by whether mothers consumed both peanuts and peanut butter or only peanut butter. For children whose mothers consumed peanuts and peanut butter while breastfeeding, higher household income was associated with increased BDNF DNAm. Additionally, maternal intake during pregnancy significantly influenced the effect of child race on IL‐6 DNAm, with divergent outcomes depending on the type of peanut product consumed.

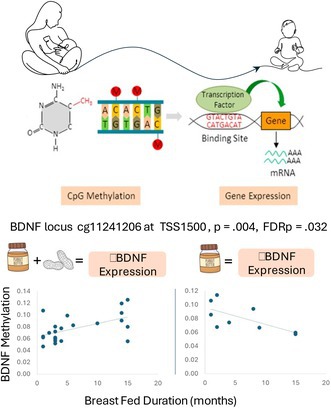

## 1 Introduction

Maternal diet during pregnancy and breastfeeding can shape both immediate and long‐term child health outcomes through nutritional, immunological, and epigenetic pathways. Certain micronutrients (e.g., folate, choline, omega‐3 fatty acids) act as methyl donors or influence inflammatory and neurodevelopmental gene expression, thereby potentially modifying DNA methylation (DNAm) patterns during critical developmental windows (Koemel and Skilton 2022; Li 2018; Pauwels et al. 2017). Poor maternal diets, such as those low in protein or high in saturated fats, have been linked to adverse epigenetic changes in offspring, affecting genes involved in metabolic regulation, inflammation, and neurodevelopment (Gali Ramamoorthy et al. 2018; Yan et al. 2017).

Breastfeeding confers both nutritional and immunological benefits as breast milk provides key nutrients for growth as well as antibodies to support immune system development (Atyeo and Alter 2021; Rogier et al. 2014; Verhasselt et al. 2024). Evidence suggests breastfeeding can also alter DNAm of genes involved in growth and energy metabolism (Briollais et al. 2021; Obermann‐Borst et al. 2013). However, the magnitude and direction of these effects may depend on maternal dietary composition during lactation, which can modify the nutrient and bioactive compound content of breast milk (Brenna et al. 2007; Cortes‐Macías et al. 2021). Breastfeeding can also impact DNAm (Hartwig et al. 2020).

Few human studies to date have evaluated how maternal dietary intake during pregnancy or breastfeeding influences epigenetic outcomes in the offspring. Little is known about whether the inclusion of specific foods during these critical periods influences offspring DNAm patterns or epigenetic changes in gene expression. Most previous research on maternal diet has focused on the development of a food allergy in the infant or child. Maternal intake of certain allergens, such as dairy, during breastfeeding has been studied in the context of short‐term tolerability by the infant related to atopic conditions or allergy (Greer et al. 2008; Kramer and Kakuma 2014; Nwaru et al. 2011). Maternal nut intake during pregnancy, once thought to be harmful, is now believed to serve as a protective factor that decreases the risk of a subsequent nut allergy in the child (Bunyavanich et al. 2014; Kramer and Kakuma 2014).

Peanuts are a nutrient‐dense, widely available food rich in protein, unsaturated fats, folic acid, choline, and polyphenols, all of which have been implicated in the epigenetic regulation of neurodevelopmental and inflammatory pathways. Most research on maternal peanut intake has focused on allergy prevention or risk (Bunyavanich et al. 2014; Kramer and Kakuma 2014), with emerging evidence suggesting that moderate maternal consumption may protect against peanut sensitization (Landau et al. 2023; Palmer et al. 2022). Far less is known about whether maternal peanut intake during pregnancy and breastfeeding influences offspring DNAm of genes related to neurodevelopment, such as brain‐derived neurotrophic factor (BDNF), or inflammation‐related genes, such as interleukin‐6 antisense RNA 1 (IL6‐AS1).

The present study investigated whether maternal peanut consumption during pregnancy and/or breastfeeding moderates associations between breastfeeding duration, household income, and child race with DNAm of BDNF and IL6‐AS1 in children aged 2–7 years. We hypothesized that maternal peanut intake would amplify these associations, revealing gene–environment interactions with potential implications for long‐term child neurodevelopment and immune regulation.

## 2 Methods

### 2.1 Procedures

This observational study recruited a convenience sample from August 2021 through August 2023 in the greater Syracuse area of NY. Inclusion criteria included children aged 2–7 years old available for in‐person data collection in Syracuse. There were no exclusion criteria. Participants were recruited using a multi‐pronged approach that included social media, targeted mailings, flyers posted at libraries, health care providers, and daycares, tabling at in‐person events, community presentations, and direct referrals from current participants. Each child and their parent or guardian participated in a single, in‐person study visit. Written informed consent was obtained from the parent/guardian, and verbal assent was obtained from the child. All procedures were approved by the Syracuse University Institutional Review Board (IRB #21‐171) and adhered to the ethical principles outlined in the Declaration of Helsinki and the Belmont Report.^1^

### 2.2 Measures

The study questionnaire included two previously validated questionnaires about the child's eating habits: Children's Eating Habits (CEHQ) (Bel‐Serrat et al. 2013) and Nutrition Screening Tool for Every Preschooler (Nutri‐STEP) (Randall Simpson et al. 2008). In addition, researchers created questions about maternal peanut and peanut butter consumption during pregnancy and breastfeeding, breastfeeding duration, the child's exposure to adverse childhood experiences (such as divorce, death in the family, witnessing a traumatic event), and demographic information (child's age and sex, mother's age, child's gestational age at birth, household income, child's race and ethnicity, and mother's education). In addition, the parent/guardian reported what the child ate the previous day using the National Cancer Institute's Automated Self‐Administered 24‐h recall (ASA‐24).

During the study visit, saliva samples were collected from children using the DNA Genotek Oragene‐Discover (OGR‐575) kit. Consent for biological samples was obtained in compliance with the Human Tissue Act (2004). Sample collection was scheduled to occur at least 1 h after the child had last eaten. Parents or guardians assisted with collection as needed, and all individuals handling samples wore latex gloves. The saliva kits include a funnel for standard collection and swabs for children unable to drool directly into the tube. Samples were stored at −80°F within 24 h and later shipped to the Diagenode lab for DNA extraction and methylation analysis using the Infinium MethylationEPIC V2.0 bead chip, which evaluates approximately 930,000 probes. A total amount of 250 ng of DNA was processed and hybridized to the Infinium MethylationEPIC V2.0 bead chip (Teschendorff et al. 2013).

### 2.3 Variables

Maternal peanut type consumption during pregnancy (MPTP) is a categorical variable indicating whether the mother consumed peanuts and peanut butter during pregnancy or peanut butter only. Maternal peanut type consumption during breastfeeding (MPTBF) is a categorical variable indicating if the mother consumed peanuts and peanut butter during breastfeeding or peanut butter only. Breastfeeding duration was measured in months as a continuous variable. Annual household income, a categorical variable, was measured in $10,000 increment categories (1–10), category 11 was $100,000–$149,999, and category 12 indicating $150,000 or more. Child race is a categorical variable with options including White/Anglo, Black/African American, Asian, American Indian or Alaska Native, Native Hawaiian or Pacific Islander, and “other” with a write‐in option. Child sex is a categorical variable (female/male). Child age is a continuous variable reported in years.

DNAm data, a continuous variable, was subset to two different methylation datasets according to the pathways associated with the targeted genes. The targeted genes BDNF and BDNF‐AS were treated as indicators of possible neurodevelopmental effects (78 CpGs). The targeted inflammation‐regulating genes were selected from previous research indicating these in association with inflammation and included CD53, HCK, IL10, IL6, IL6‐AS1, IL7, TYROBP (83 CpGs).

### 2.4 Statistical Analyses

#### 2.4.1 Covariates

Covariates were selected a priori based on prior evidence linking these variables to variation in DNAm profiles in pediatric cohorts, as well as their potential to confound associations between maternal diet, breastfeeding duration, and DNAm. These included child sex, child race, epithelial cell proportion, maternal age at child's birth, and household income. Each has been associated with DNAm in prior studies and/or with the exposure or outcome variables in this analysis. Controlling for these factors helps isolate the effects of maternal peanut intake and breastfeeding duration on DNAm patterns.

#### 2.4.2 DNA Methylation

An initial data quality assessment was performed using the R package *CpGassoc* (Barfield et al. 2012). CpG sites exhibiting negligible signal or with more than 10% missing data across samples were removed, as were samples missing more than 5% of CpG sites. Cross‐reactive probes were excluded (Zhang et al. 2022). Beta values (*β*) for each CpG site were calculated as the ratio of the methylated (M) signal to the sum of the methylated and unmethylated (*M* + *U*) signals: *β* = *M*/(*M* + *U*). Quantile normalization was performed as previously described by Teschendorff et al. (2013).

#### 2.4.3 Cell‐Type Estimation and Batch Correction

The proportion of epithelial cells in saliva was estimated using the Robust Partial Correlation (RPC) method implemented in *EpiDISH* (Zheng 2024) with the centEpiFibIC reference data (Zheng et al. 2018). Batch effects related to chip and position were corrected using the ComBat method (Johnson et al. 2007).

#### 2.4.4 Statistical Modeling

A missing value analysis confirmed no missing data for phenotype variables. Linear regression models tested direct associations of maternal peanut type consumption during pregnancy (MPTP) and breastfeeding (MPTBF), breastfeeding duration, household income, and child race with DNAm of BDNF/BDNF‐AS (78 CpGs) and inflammation‐related genes (83 CpGs).

Moderation was assessed using interaction terms (e.g., MPTP × breastfeeding duration) as predictors, with DNAm as the outcome. All models included the covariates listed above, as well as the independent terms comprising each interaction. The False Discovery Rate (Storey 2011) controlled for multiple comparisons, with adjusted *p*‐values reported as FDRp. Significant associations were visualized for interpretation.

## 3 Results

### 3.1 Cohort

Child participants in our study (*n* = 35) were aged 2 to 7 years (4.1 ± 1.0 years) and 54% male. Child race (as identified by the parent/guardian) was White/Anglo (51.4%), Black/African American (25.7%), and biracial (22.9%; Table 1). Ten children were identified as Latino/Hispanic. A total of 34% of participants reported $10,000 or less in annual household income. Breastfeeding duration ranged from < 1 month up to 15 months (6.06 ± 5.25 months). During pregnancy, 70.9% of mothers consumed peanuts and peanut butter. During breastfeeding, 63.2% of mothers consumed peanuts and peanut butter.

**TABLE 1:** Participant characteristics and summary of direct effects on child DNAm.

Characteristic	Value
Sample size (*n*)	35
Child age (years)	2–7 years; mean ± SD = 4.1 ± 1.0
Child sex	54% male
Child race	White/Anglo: 51.4%
	Black/African American: 25.7%
	Biracial: 22.9%
Ethnicity	Latino/Hispanic: 10 children
Annual household income	≤ $10,000: 34% of participants
Breastfeeding duration (months)	Range: < 1–15 months; mean ± SD = 6.06 ± 5.25
Maternal peanut & peanut butter intake during pregnancy (MPTP)	70.9%
Maternal peanut & peanut butter intake during breastfeeding (MPTBF)	63.2%
Direct effects on DNAm	
Breast feeding duration	FDRp > 0.05
Household income	FDRp > 0.05
MPTP	FDRp > 0.05
MPTBF	FDRp > 0.05

Breastfeeding duration (FDRp > 0.05), household income (FDRp > 0.05), MPTP (FDRp > 0.05), and MPTBF (FDRp > 0.05) did not show direct effects on child DNAm of BDNF, BDNF‐AS, or inflammation‐regulating genes. However, several moderating effects were observed between these predictor variables and child DNAm of the targeted genes.

### 3.2 BDNF and BDNF‐AS Child DNAm Moderation

#### 3.2.1 Breastfeeding Duration

Maternal peanut type consumption during pregnancy significantly moderated the relationship between breastfeeding duration and child DNAm at 18 CpG sites within the BDNF and BDNF‐AS genes. All sites remained significant after false discovery rate (FDR) correction (FDRp = 0.032–0.049).

**FIGURE 1:**
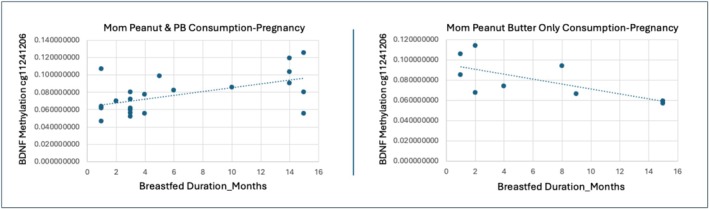
Maternal peanut product type consumption during pregnancy moderates the effects of breastfeeding. MPTP moderates the association between breastfeeding duration and child BDNF DNAm of BDNF cg11241206 located at TSS1500 on the S shore (*B* = 0.06, *t* = 3.206, *p* = 0.004, FDRp = 0.032). Mothers who consumed peanuts and peanut butter *during pregnancy* had children who showed increased DNAm of BDNF cg11241206 as breastfeeding duration increased (*R* ^2^ = 0.296). Whereas mothers who consumed peanut butter only during pregnancy had children who showed decreased DNAm of BDNF cg11241206 as breastfeeding duration increased (*R* ^2^ = 0.462).

**FIGURE 2:**
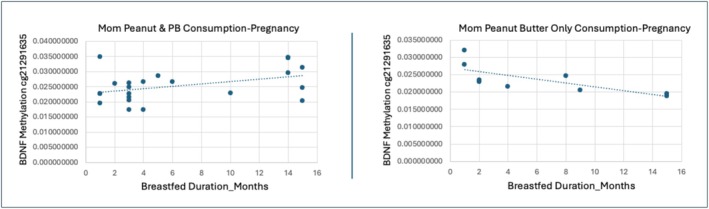
Maternal peanut product type consumption during pregnancy moderates breastfeeding effects. MPTP moderates the association between breastfeeding duration and child DNAm of BDNF cg21291635 located at TSS1500 on the island (*B* = 0.042, *t* = 3.368, *p* = 0.002, FDRp = 0.032). Maternal consumption of peanuts and peanut butter during pregnancy shows increased child DNAm of BDNF cg21291635 as breastfeeding duration increases (*R* ^2^ = 0.167). Maternal consumption of peanut butter only during pregnancy shows decreased child DNAm of BDNF cg21291635 as breastfeeding duration increases (*R* ^2^ = 0.547).

The majority of CpG sites (12 in BDNF and 3 in BDNF‐AS) showed increased methylation with longer breastfeeding duration among children whose mothers consumed both peanuts and peanut butter during pregnancy, compared to those whose mothers consumed peanut butter only (Figures 1 and 2). Three BDNF CpG sites exhibited decreased methylation under the same conditions (Figures 1 and 2). Functionally, most of the significant CpG sites were located in transcription start site regions (TSS1500/TSS200), 5′ untranslated regions (5′UTR), or exons, with 16 of the 18 sites positioned in or near CpG islands or shores—genomic contexts associated with transcriptional regulation. Full statistical results for these CpG sites, including t‐statistics, coefficients, standard errors, and genomic annotations, are provided in Table 2.

**TABLE 2:** Significant CpG sites for the interaction between maternal peanut type consumption during pregnancy (MPTP) and breastfeeding duration on child BDNF/BDNF‐AS DNAm (*n* = 35).

CpG ID	Gene name	Genomic feature(s)	Relation to Island	*t*‐statistic	Coefficient	SE	*p*	FDR
cg11241206	BDNF	5′UTR; exon_1; TSS1500	S_Shore	3.21	0.0626	0.0195	0.0041	0.0322
cg16141574	BDNF	exon_2–4; exon_1	N/A	−3.75	−0.0571	0.0152	0.0011	0.0322
cg16201963	BDNF‐AS	TSS1500	Island	3.20	0.0862	0.0269	0.0041	0.0322
cg20144981	BDNF‐AS	TSS1500	N_Shore	3.39	0.0810	0.0239	0.0027	0.0322
cg21291635	BDNF	5′UTR; exon_1; TSS1500	Island	3.37	0.0425	0.0126	0.0028	0.0322
cg22288103	BDNF	TSS200; TSS1500	Island; S_Shelf; S_Shore	3.28	0.0570	0.0174	0.0034	0.0322
cg22830701	BDNF‐AS	TSS1500	Island	3.35	0.0898	0.0268	0.0030	0.0322
cg23426002	BDNF	exon_2–4; exon_1	N/A	−3.33	−0.0593	0.0178	0.0030	0.0322
cg25381667	BDNF	TSS200; TSS1500	Island; S_Shelf; S_Shore	3.23	0.0707	0.0219	0.0039	0.0322
cg27351358	BDNF	exon_1; 5′UTR; TSS1500	S_Shelf; S_Shore; N_Shore	3.36	0.0781	0.0232	0.0028	0.0322
cg01225698	BDNF	TSS1500; TSS200	S_Shore; N_Shore	2.94	0.0664	0.0226	0.0075	0.0420
cg06046431	BDNF	TSS1500	S_Shelf; Island	3.04	0.0322	0.0106	0.0060	0.0420
cg15688670	BDNF	TSS1500; TSS200	S_Shore	2.98	0.0510	0.0171	0.0069	0.0420
cg26057780	BDNF	TSS200	N_Shore	2.97	0.0469	0.0158	0.0070	0.0420
cg03747251	BDNF	TSS1500; TSS200	S_Shore	2.88	0.0702	0.0244	0.0087	0.0428
cg17882499	BDNF	TSS1500; TSS200	Island	−2.88	−0.0239	0.0083	0.0088	0.0428
cg25412831	BDNF	5′UTR; exon_1; TSS1500	S_Shore; Island; N_Shore	2.81	0.0732	0.0261	0.0103	0.0471
cg07159484	BDNF	exon_1; 5′UTR; TSS1500; TSS200	Island	2.77	0.0709	0.0256	0.0113	0.0488

#### 3.2.2 Household Income and Child Race

**FIGURE 3:**
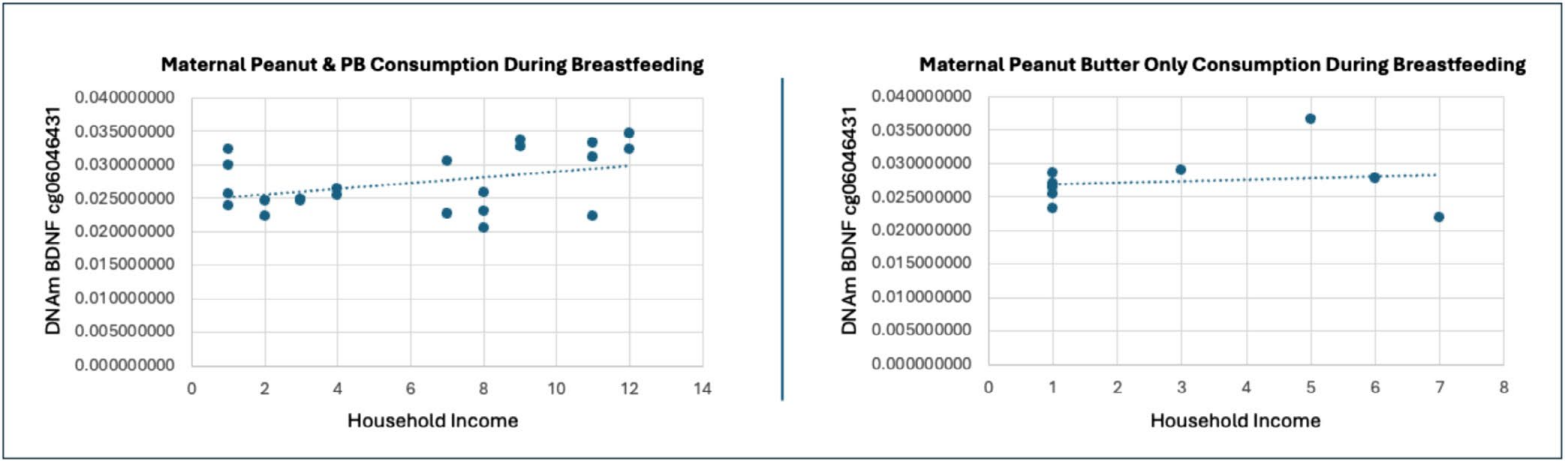
Maternal peanut product type consumption during breastfeeding moderation of income effects. MPTBF shows a moderating effect of income association with child DNAm of BDNF cg06046431 located at TSS1500 on the S_shore in offspring (*B* = 0.058, *t* = 4.862, *p* = 0.0005, FDRp = 0.039). Maternal consumption of peanut and peanut butter while breastfeeding increases child DNAm of BDNF cg06046431 as household income increases (*R* ^2^ = 0.151). Maternal peanut butter only consumption while breastfeeding doesn't show a clinically relevant or statistically significant effect on child DNAm of BDNF cg06046431 as household income increases (*R* ^2^ = 0.017).

MPTBF moderates household income associations with offspring DNAm of BDNF on one CpG site cg06046431 located at TSS1500 on the S_shore in offspring (*B* = 0.058, *t* = 4.862, *p* = 0.0005, FDRp = 0.039) shown in Figure 3. MPTP did not show an interaction with household income in association with child DNAm of BDNF and BDNF‐AS (FDRp > 0.05). Lastly, MPTP and MPTBF were not indicated for interactions with child race in association with child DNAm of BDNF and BDNF‐AS (FDRp > 0.05).

### 3.3 Inflammation Regulating Genes Child DNAm

#### 3.3.1 Household Income and Child Race

MPTBF was not indicated for a moderating interaction with income for inflammation regulating gene DNAm (FDRp > 0.05). Covariates in the model include child sex, child race, cell type, maternal age at birth, and both interaction terms maternal peanut type consumption during breastfeeding and household income. MPTP also did not show an interaction with household income associations with inflammation regulating genes (FDRp > 0.05). MPTP and MPTBF did not show interactions with breastfeeding duration in association with genes regulating inflammation (FDRp > 0.05).

**FIGURE 4:**
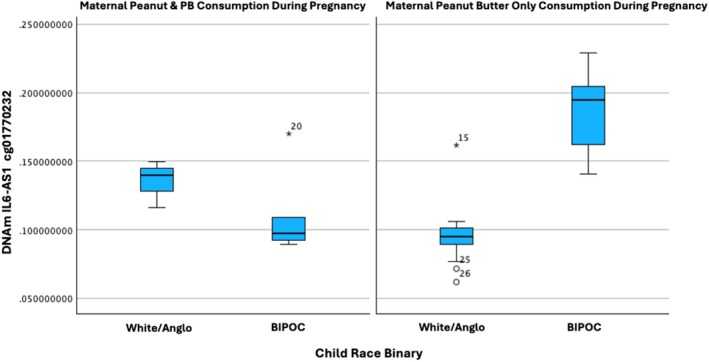
Child race moderation of MPTP effects on inflammation genes. Child race was dichotomized to increase group size comparison. Children identified as Black, Latino/Latina, or Bi‐racial were condensed into a BIPOC (Black, indigenous, people of color) group given similar marginalization experienced by these populations that increases stress effects that could influence DNAm of inflammation regulating genes. MPTP moderates the race association with DNAm of *IL6‐AS1* cg01770232 on chromosome 7, indicated as an enhancer, located at TSS1500 (*B* = 1.161, *t* = 5.577, *p* = 1.128 × 10^−5^, FDR*p* = 0.0009). The BIPOC group of children showed reduced DNAm of *IL6‐AS1* cg01770232 with mothers who consumed peanuts and peanut butter during pregnancy. Maternal peanut butter only consumption during pregnancy showed children with increased DNAm of *IL6‐AS1* cg01770232 in the BIPOC group. *IL6‐AS1* cg01770232 is an opposite strand CpG site.

MPTBF moderates an association between child race and one CpG site of the IL6‐AS1 cg01770232 on chromosome 7, indicated as an enhancer, located at TSS1500 (*B* = 1.161, *t* = 5.577, *p* = 1.128 × 10^−5^, FDRp = 0.0009). Among BIPOC children, maternal consumption of both peanuts and peanut butter during pregnancy was associated with reduced DNA methylation at IL6‐AS1 cg01770232. In contrast, when mothers consumed only peanut butter during pregnancy, BIPOC children exhibited increased DNA methylation at this same site. The interaction is modeled in Figure 4 for interpretation. MPTP was not indicated for an interaction with child race in association with inflammation regulating genes (FDRp > 0.05).

## 4 Discussion

This study is the first, to our knowledge, to identify dynamic moderating effects of maternal peanut and peanut butter consumption during pregnancy and breastfeeding on the relationships between breastfeeding duration and patterns of DNAm of neurodevelopment and inflammation regulating genes in children. Our findings provide novel evidence that early nutritional exposures, particularly the form of peanut intake, may shape epigenetic programming during a critical window of neurodevelopment.

Our findings may also be interpreted in light of the dual allergen exposure hypothesis, which proposes that early oral exposure to allergenic proteins promotes immune tolerance, while environmental (non‐oral) exposure in the absence of oral intake may promote sensitization (Lack 2012). Several studies have demonstrated that maternal dietary inclusion of allergenic foods such as peanuts during pregnancy and breastfeeding can reduce the risk of subsequent food allergy in the child (Fox et al. 2009; Azad et al. 2021; Palmer et al. 2022; Landau et al. 2023). Mechanistically, allergen shedding into breast milk (Macchiaverni et al. 2021) provides repeated low‐dose oral exposure to the infant, which may train the developing immune system and promote tolerance. In our study, differences in IL6‐AS1 DNAm patterns between “peanuts + peanut butter” and “peanut butter only” groups—particularly among BIPOC participants—could reflect variations in both the amount and allergenic potential of peanut proteins transferred through breast milk. Processing methods for peanut butter, which can reduce allergenicity (Salve et al. 2021), may also contribute to the divergent epigenetic profiles observed. Together, these findings underscore the importance of considering both the form of maternal allergen consumption and potential oral pathways when interpreting diet–epigenetic associations.

In our study, maternal peanut type consumption during pregnancy moderated the effect of breastfeeding duration on child DNAm of BDNF and BDNF‐AS. Among participants whose mothers consumed both peanuts and peanut butter during pregnancy, longer breastfeeding duration was associated with increased DNA methylation at 12 BDNF and 3 BDNF‐AS CpG sites. In contrast, 3 BDNF CpG sites showed decreased methylation, with only one of these located at a transcription start site (TSS). This pattern suggests that the observed decreases in methylation are less likely to impact transcriptional regulation or downstream expression of the BDNF gene, as methylation at TSSs is more directly linked to gene silencing. Overall, our results suggest that in general, maternal consumption of both peanuts and peanut butter during pregnancy enhances the epigenetic sensitivity of the BDNF gene region to breastfeeding duration. This nutrient–epigenetic interaction is evidence that maternal diet (in this case, specific peanut products) may prime the infant's epigenome, making the duration of breastfeeding more influential on the child's neurodevelopmental gene regulation.

Supporting this interpretation, the majority of hypermethylated sites were located at transcription start sites and within CpG islands or shores, genomic regions known to play a key role in the regulation of gene expression. In contrast, only one of the hypomethylated CpG sites was in a promoter region, suggesting a greater likelihood of functional downregulation via reduced BDNF transcription for the hypermethylated sites. This finding is intriguing, given that both breastfeeding and peanut polyphenol intake have been associated with enhanced neurocognitive outcomes. For example, breastmilk has been previously found to contain BDNF (Martysiak‐Żurowska et al. 2021), and polyphenols in peanuts have been linked to cognitive function and are theorized to affect BDNF pathways (Parilli‐Moser et al. 2021). One possible explanation for our observation is that the combination of breastfeeding and greater consumption of polyphenols through peanuts and peanut butter during pregnancy increases serum brain‐derived neurotrophic factors in offspring to a degree that triggers epigenetic downregulation of the BDNF gene. Further research examining BDNF serum levels in relation to maternal peanut product consumption during pregnancy and breastfeeding interactions could clarify this relationship.

For participants with maternal consumption of peanut butter only during pregnancy, longer breastfeeding was associated with decreased BDNF methylation, suggesting enhanced downstream gene expression potential. Past research has found no effect of exclusive breastfeeding (compared to formula feeding or a mix of feeding types) on DNAm of BDNF at 2 years of age (Cheshmeh et al. 2022). Similarly, among a cohort of 8–11‐year‐old children, serum levels of BDNF were not linked to breastfeeding status during infancy (Berlanga‐Macías et al. 2021). In both prior studies, maternal diet during pregnancy or breastfeeding was not reported or accounted for in the analysis. This is an important consideration for future studies, as our results suggest that specific maternal intakes may influence the direction of the relationship between breastfeeding duration and DNAm of BDNF.

Our findings also suggest socioeconomic context interacts with maternal diet to shape epigenetic profiles in a similarly divergent manner. When mothers ate both peanuts and peanut butter during breastfeeding, household income positively predicted DNAm at a BDNF CpG site near a transcription start site. This may indicate that more favorable environmental conditions (e.g., higher income) combined with specific dietary exposures further promote BDNF downregulation through methylation. However, no such interaction was observed among participants whose mothers consumed peanut butter only during breastfeeding, and no significant interactions with income were observed for inflammation‐related genes.

While income and maternal peanut type consumption did not associate with inflammation regulating genes, a race interaction did emerge. BIPOC participants showed reduced DNAm of IL6‐AS1 cg01770232 compared to white peers when mothers consumed both peanuts and peanut butter during pregnancy, but increased DNAm when mothers consumed peanut butter only during pregnancy. The opposite effects observed could be due to changes in allergens in peanut butter due to the processing that takes place to make peanut butter. For example, thermal processing of peanuts, which commonly occurs in making peanut butter, has been shown to reduce the allergenicity of peanuts (Salve et al. 2021). The reduced allergen potential could explain the increased DNAm of IL6‐AS1 as a possible downregulation of the inflammation regulating gene increasing the prospect of a reduced inflammatory response. This is critical given that inflammation is a known allergic response (Cheng et al. 2023; Martin et al. 2024; Small et al. 2018). The lack of moderating associations for race with BDNF could be due to a true absence of association or due to type II error from reduced statistical power from a small sample size. Future research would benefit from examining this race relationship further with a larger diverse sample to explore whether differences may be due to oxidative stress resulting from discrimination experiences and systemic oppression issues.

Our study does have some limitations. Detailed information on maternal peanut and peanut butter consumption, such as frequency and typical serving size, was not collected in this study. In addition, we did not ask about prenatal or postpartum supplement (multivitamin) use. As a result, we are unable to pinpoint exact nutrients that may be responsible for the pattern of our results. Despite this, peanuts are known to have a higher content of folic acid than peanut butter when comparing typical portion sizes (68 mcg in 1 oz. peanuts vs. 11 mcg in 2 Tbsp. peanut butter). This increased dose of folic acid may be enough to cause greater differences in DNAm to explain our study observations. Future research should more precisely quantify maternal intake of peanuts versus peanut butter during pregnancy, including portion sizes and frequency, to clarify the specific nutrient contributions to observed differences in child DNAm patterns.

## 5 Conclusion

Breastfeeding duration, household income, and race have all been identified as factors that can influence child development by contributing either protective benefits or increased risks (Bernard et al. 2013; Cooper and Stewart 2021; Henry et al. 2019). The key findings from this study show that maternal nutrition factors, specifically peanut type consumption, during pregnancy and breastfeeding have moderating effects on genes that regulate neurodevelopment and inflammation in young children. Future research would benefit from continued exploration of how maternal nutrition during pregnancy and breastfeeding may moderate the effects of breastfeeding, household income, and race on child development.

## Author Contributions

**Jessica L. Garay:** conceptualization (equal), data curation (lead), formal analysis (supporting), funding acquisition (lead), investigation (lead), methodology (equal), project administration (lead), resources (equal), supervision (equal), writing – original draft (equal). **Margaret A. Voss:** conceptualization (supporting), formal analysis (supporting), validation (supporting), writing – review and editing (equal). **Stefanie R. Pilkay:** conceptualization (equal), data curation (supporting), formal analysis (lead), funding acquisition (supporting), investigation (equal), methodology (equal), writing – original draft (supporting).

## Conflicts of Interest

The authors declare no conflicts of interest.

## Supporting information


**Figure S1:** Maternal peanut product type consumption during pregnancy moderation of breastfeeding effects.
**Figure S2:** Maternal peanut product type consumption during pregnancy moderation of breastfeeding effects.
**Table S1:** Questions and response options for maternal diet, breastfeeding, and adverse childhood experiences.

## Data Availability

The data that support the findings of this study are available from the corresponding author upon reasonable request.

## Endnotes

^1^
This study was approved by the Syracuse University Institutional Review Board (IRB #21–171). Written informed consent was obtained from all study participants.
