# Supplementary material for: Effects of Maternal Peanut Intake and Breastfeeding Duration on Offspring DNA Methylation
Source: Food Sci Nutr. 2025 Oct 21;13(10):e71129. doi: 10.1002/fsn3.71129 (PMC12540189; doi:10.1002/fsn3.71129)
Supplement: Supplementary file 1 — Figure S1: Maternal peanut product type consumption during pregnancy moderation of breastfeeding effects. Figure S2: Maternal peanut product type consumption during pregnancy moderation of breastfeeding effects. Table S1: Questions and response options for maternal diet, breastfeeding, and adverse childhood experiences. [file FSN3-13-e71129-s001.docx]

**Supplemental Figure 1.**

**Maternal Peanut Product Type Consumption During Pregnancy Moderation of Breastfeeding Effects**


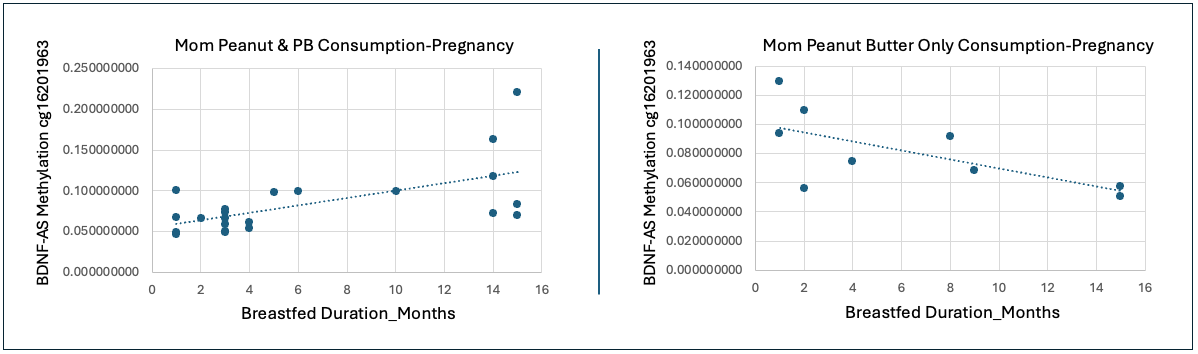


Note. MPTP moderates association between breastfeeding duration and child DNAm of BDNF-AS cg16201963 located at TSS1500 on the island (B = .086, t = 3.200, p = .004, FDRp = .032). Mothers who consumed peanuts and peanut butter during pregnancy had children who showed increased DNAm of BDNF-AS cg16201963 as breastfeeding duration increased (R^2^ = .373). Yet, mothers who consumed peanut butter only during pregnancy had children who showed reduced DNAm of BDNF-AS cg16201963 as breastfeeding duration increased (R^2^ = .438).

**Supplemental Figure 2.**

**Maternal Peanut Product Type Consumption During Pregnancy Moderation of Breastfeeding Effects**


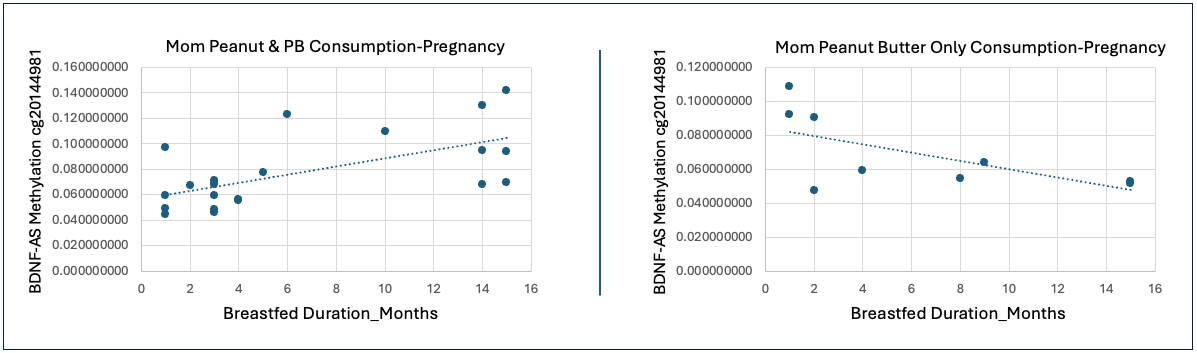


Note. MPTP moderates association between breastfeeding duration and child DNAm of BDNF-AS cg20144981 located at TSS1500 on the N_Shore in offspring (B = .081, t = 3.386, p = .002, FDRp = .032). Maternal consumption of peanuts and peanut butter during pregnancy shows increased child DNAm of BDNF-AS cg20144981 as breastfeeding duration increases (R^2^ = .382). Maternal consumption of peanut butter only during pregnancy shows decreased DNAm of BDNF-AS cg20144981 in offspring as breastfeeding duration increases (R^2^ = .401).

**Supplemental Table 1.**

**Questions and response options for maternal diet, breastfeeding, and adverse childhood experiences**

1. We are interested in knowing the birth weight and birth length of your child.

- Birth weight (please enter the amount of **pounds** your child weighed):
- Birth weight (please enter the amount of **ounces** your child weighed):
- Birth length (please enter the number of **inches**):

1. Enter your child's due date as MM/DD/YYYY:
2. Enter your child's actual birth date as MM/DD/YYYY:
3. How long was your child breastfed?
   1. None
   2. 1 week or less
   3. 1 month or less
   4. 2 months
   5. 3 months
   6. 4 months
   7. 5 months
   8. 6 months
   9. 7 months
   10. 8 months
   11. 9 months
   12. 10 months
   13. 11 months
   14. 12 months
   15. More than 1 year
4. Did your child use infant formula?
   1. Yes (Move to Question 6)
   2. No (Move to Question 9)
5. At what age did you start using infant formula for your child?
   1. Immediately at birth
   2. Within the first week
   3. Within the first month
   4. 2 months old
   5. 3 months old
   6. 4 months old
   7. 5 months old
   8. 6 months old
   9. 7 months old
   10. 8 months old
   11. 9 months old
   12. 10 months old
   13. 11 months old
   14. 12 months old
   15. More than 1 year old
6. Why did you begin using infant formula? You can select more than one answer if needed.

- Pediatrician recommended it for weight gain or other issue.
- My child was having reflux or digestive issues from breastmilk.
- I wasn't producing enough breastmilk.
- I was ready to stop breastfeeding.
- I was given free samples, coupons, or vouches for infant formula.
- Other (please specify):

1. When you began using infant formula, did your child receive:

- A mixture of breastmilk + infant formula
- Only infant formula
- Other (please describe below):

1. When did your child begin eating solid food? This includes rice cereal, teething crackers, etc.
   1. Within the first month
   2. 2 months old
   3. 3 months old
   4. 4 months old
   5. 5 months old
   6. 6 months old
   7. 7 months old
   8. 8 months old
   9. 9 months old
   10. 10 months old
   11. 11 months old
   12. 12 months old
   13. More than 1 year old
2. What was the first solid food your child began eating? You can select more than one answer.

- Rice cereal
- Teething crackers
- Baby food from a jar
- Baby food from a squeeze pouch
- Homemade baby food
- Small pieces of food I was eating
- Other (please describe):

1. Did the child's mother consume peanuts or peanut butter during her pregnancy with the child?

- Yes - both peanuts and peanut butter
- Yes - only peanuts
- Yes - only peanut butter
- No
- Don't know

1. Did the child's mother consume peanuts or peanut butter while breastfeeding the child?

- Yes - both peanuts and peanut butter
- Yes - only peanuts
- Yes - only peanut butter
- No
- Don't know

1. At what age did your child first eat peanut butter?
2. Within the first month
3. 2 months old
4. 3 months old
5. 4 months old
6. 5 months old
7. 6 months old
8. 7 months old
9. 8 months old
10. 9 months old
11. 10 months old
12. 11 months old
13. 12 months old
14. As a 1 year old
15. As a 2 year old
16. As a 3 year old
17. As a 4 year old
18. At what age did your child first eat peanuts?
19. Within the first month
20. 2 months old
21. 3 months old
22. 4 months old
23. 5 months old
24. 6 months old
25. 7 months old
26. 8 months old
27. 9 months old
28. 10 months old
29. 11 months old
30. 12 months old
31. As a 1 year old
32. As a 2 year old
33. As a 3 year old
34. As a 4 year old
35. Has the child been diagnosed with any food allergies? Please select all that apply.

- Peanut
- Milk
- Egg
- Fish/Shellfish
- Soy
- Other (please write in):

1. Since your child has been born, who has been responsible for providing childcare for your child? Use the table below to indicate for each age of your child. You can select more than one provider for each age.

|  | A biological parent | A close relative (i.e. grandparent) | Day care at someone else's home | Day care center | Head Start program | Other |
| --- | --- | --- | --- | --- | --- | --- |
| When the child was an infant (under 1 year) |  |  |  |  |  |  |
| 1-2 years old |  |  |  |  |  |  |
| 2-3 years old |  |  |  |  |  |  |
| 3-4 years old |  |  |  |  |  |  |

If you selected "Other" in the question above, please explain who provided day care for your child:

The following questions were asked of the parent/guardian.

1. What is your current age (in years)?
2. What is the highest level of school you have completed or the highest degree you have received?

- Less than high school degree
- High school graduate (high school diploma or equivalent including GED)
- Some college but no degree
- Associate degree in college (2-year)
- Bachelor's degree in college (4-year)
- Master's degree
- Doctoral degree
- Professional degree (JD, MD)

1. Are you Spanish, Hispanic, or Latino or none of these?
   1. Yes (go to Question 20)
   2. None of these (go to Question 21)
2. Are you Spanish, Hispanic, or Latino?

- Spanish
- Hispanic
- Latino

1. Choose one or more races that you consider yourself to be:

- White
- Black or African American
- American Indian or Alaska Native
- Asian
- Native Hawaiian or Pacific Islander
- Other:

1. What is your sex?
   1. Male
   2. Female
2. Information about income is very important to understand.  Would you please give your best guess? Please indicate the answer that includes your entire household income in (previous year) before taxes.

- Less than $10,000
- $10,000 to $19,999
- $20,000 to $29,999
- $30,000 to $39,999
- $40,000 to $49,999
- $50,000 to $59,999
- $60,000 to $69,999
- $70,000 to $79,999
- $80,000 to $89,999
- $90,000 to $99,999
- $100,000 to $149,999
- $150,000 or more

1. What is your ZIP code?
2. Since your child was born, has he or she lived with anyone who went to prison?
   1. Yes
   2. No
3. Has your child experienced any of the following situations? Please select all that may apply.

- Parents have divorced or broke up
- One or both parents have abandoned child
- One or both parents have died
- A sibling or other close relative has died
- A parent, sibling, or close relative has attempted suicide
- Witnessed a traumatic event (i.e. accident, fire, abuse)
- Moved to a new location

1. Since the child has been born, has there ever been a situation where the child, or the household overall, has not had enough food to eat?
   1. Yes
   2. No
2. How often does your child usually eat at home or at other people’s home (e.g. grandparents, friends)? Please select an answer for every meal.

|  | Daily | Only on weekdays | Only on weekends | Several times per week | On fewer occasions |
| --- | --- | --- | --- | --- | --- |
| Breakfast |  |  |  |  |  |
| Lunch |  |  |  |  |  |
| Dinner |  |  |  |  |  |
| Snacks |  |  |  |  |  |

1. How often does your child eat while doing something else (e.g. watching TV, playing, sitting at a computer, looking at a book)?

- Never, or rarely
- Several times per week
- Once a day
- On several occasions per day

1. Does your child normally avoid eating any of the following foods?

|  | Yes | No |
| --- | --- | --- |
| Meat, poultry, or sausage |  |  |
| Fish |  |  |
| Milk and milk products |  |  |
| Eggs |  |  |
| Other (please indicate): |  |  |

1. How many times does your child eat fast food in a fast food restaurant...

|  | Never | Once a month or less | Several times a month | 1-2 times a week | 3 or more times a week |
| --- | --- | --- | --- | --- | --- |
| to consume a full meal alternative to a normal meal (breakfast, lunch dinner)? |  |  |  |  |  |
| to consume some food as snack between meals? |  |  |  |  |  |
